# Elevated temperature alters microbial communities, but not decomposition rates, during 3 years of *in situ* peat decomposition

**DOI:** 10.1128/msystems.00337-23

**Published:** 2023-10-11

**Authors:** Spencer W. Roth, Natalie A. Griffiths, Randall K. Kolka, Keith C. Oleheiser, Alyssa A. Carrell, Dawn M. Klingeman, Angela Seibert, Jeffrey P. Chanton, Paul J. Hanson, Christopher W. Schadt

**Affiliations:** 1 Biosciences Division, Oak Ridge National Laboratory, Oak Ridge, Tennessee, USA; 2 Environmental Sciences Division, Oak Ridge National Laboratory, Oak Ridge, Tennessee, USA; 3 Climate Change Science Institute, Oak Ridge National Laboratory, Oak Ridge, Tennessee, USA; 4 Northern Research Station, USDA Forest Service, Grand Rapids, Minnesota, USA; 5 Department of Geosciences, Boise State University, Boise, Idaho, USA; 6 Department of Earth, Ocean, and Atmospheric Science, Florida State University, Tallahassee, Florida, USA; 7 Department of Microbiology, University of Tennessee, Knoxville, Tennessee, USA; Pacific Northwest National Laboratory, Richland, Washington, USA

**Keywords:** peatlands, climate change, microbiome, organic matter decomposition

## Abstract

Peatlands are large carbon sinks with primary production outpacing decomposition of organic matter. Results from the Spruce and Peatland Responses Under Changing Environments (SPRUCE) study show net losses of organic matter and increased greenhouse gas production from peatlands in response to whole-ecosystem warming. Here, we investigated how warming and elevated CO_2_ impact peat microbial communities and peat soil decomposition rates and characterized microbial communities through amplicon sequencing and compositional changes across four depth increments. Microbial diversity and community composition were significantly impacted by soil depth, temperature, and CO_2_ treatment. Bacterial/archaeal α-diversity increased significantly with increasing temperature, and fungal α-diversity was lower under elevated CO_2_ treatments. *Trans* domain microbial networks showed higher complexity of microbial communities in decomposition ladder depths from the warmed enclosures, and the number of highly connected hub taxa within the networks was positively correlated with temperature. Methanogenic hubs were identified in the networks constructed from the warmest enclosures, indicating increased importance of methanogenesis in response to warming. Microbial community responses were not however reflected in measures of peat soil decomposition, as warming and elevated CO_2_ had no significant short-term effects on soil mass loss or composition. Regardless of treatment, on average only 4.5% of the original soil mass was lost after 3 years and variation between replicates was high, potentially masking treatment effects. Previous results at the SPRUCE experiment have shown warming is accelerating organic-matter decomposition and CO_2_ and CH_4_ production, and our results suggest these changes may be driven by warming-induced shifts in microbial communities.

**IMPORTANCE:**

Microbial community changes in response to climate change drivers have the potential to alter the trajectory of important ecosystem functions. In this paper, we show that while microbial communities in peatland systems responded to manipulations of temperature and CO_2_ concentrations, these changes were not associated with similar responses in peat decomposition rates over 3 years. It is unclear however from our current studies whether this functional resiliency over 3 years will continue over the longer time scales relevant to peatland ecosystem functions.

## INTRODUCTION

Despite covering less than 10% of the Earth’s surface, peatlands contain approximately one-third of all global terrestrial organic matter (OM) (1 – 3). Peatland organic soil deposits can be several meters deep—the result of thousands of years of net primary production outpacing OM mineralization. Microorganisms are primarily responsible for OM degradation in peatlands (4, 5); however, anoxic, acidic, oligotrophic, and cold conditions that are common across northern peatlands greatly constrain microbial activity (4).

Climate change has the potential to alter peatland biogeochemistry, especially at northern latitudes where warming is occurring at an accelerated pace compared to equatorial regions (6). The large carbon stocks in northern peatlands that have built up over millennia may therefore be vulnerable to climate change (7); however, the effects of warming and increased atmospheric CO_2_ on peatland ecosystems remain to be fully described (8, 9). The Spruce and Peatland Responses Under Changing Environments (SPRUCE) experiment is a long-term warming and elevated CO_2_ experiment investigating peatland responses to climate change on an ecosystem level (https://mnspruce.ornl.gov/). Since 2016, whole-ecosystem warming up to +9°C above ambient has been applied to a boreal peatland in a regression-based design. In addition, elevated air partial pressure of CO_2_ has been applied to half of the 10 SPRUCE experimental enclosures.

Results from the SPRUCE experiment have shown significant, rapid loss of carbon from the peatland with increasing temperature (10) concomitant with a large decline and death of *Sphagnum* sp. at the highest temperatures, which contribute the largest share of gross primary production (GPP) in these ecosystems (11). In addition, porewater concentrations of CO_2_ and CH_4_ have been shown to correlate with temperature treatment at SPRUCE (12), and radiocarbon analysis of soil suggests that warming is promoting microbial respiration of solid-phase peat (13). Similar results have also been obtained in studies of other peatlands (14 – 16) and in incubation studies that show increasing CH_4_ and CO_2_ production with warming (17, 18).

Net losses of carbon from the SPRUCE sites have been attributed to increased degradation of OM, rather than a reduction in primary production (10). Under anoxic conditions, microbial degradation of OM involves multiple steps including hydrolysis, fermentation, and anaerobic respiration. Mineralization of carbon to CO_2_ and CH_4_ in peatlands therefore relies on microbial metabolic interactions that may be altered by climate change. Incubation experiments have demonstrated that microbial activities (19, 20) and metabolic interactions are significantly altered by warming (15), although impacts on community structure and diversity vary (17, 18, 21, 22). *In situ* results showing increasing CH_4_:CO_2_ ratios with warming suggest that microbial interactions in the SPRUCE sites may be altered to favor increased methanogenesis (12, 23).

Previous studies have investigated the effects of warming on peat soil decomposition through incubations or whole-ecosystem assessments (10, 12, 13, 17, 18, 24). Valuable insights have been gained from these experiments; however, incubation studies do not fully reflect environmental conditions, and “bottle effects” may influence microbial community structure (25). Conversely, assessing decomposition from *in situ* environmental measurements is complex and may be influenced by other ecosystem processes such as changes in primary productivity. To overcome these limitations, we utilized new peat soil decomposition ladders, which are peat litter bags attached to a rigid frame, to assess the impacts of temperature and CO_2_ treatments on peat soil decomposition at four depths. This approach allows for *in situ* investigation of decomposition while controlling for the effects of primary productivity and excluding fresh litter inputs. While studies of fresh plant litter decomposition using similar methods are quite common across many forest ecosystem types (26), studies of soil residue and particularly peat decomposition using decomposition bag methods appear to be absent from the literature. Peat decomposition ladders were deployed in the top 40 cm of peat in the 10 SPRUCE experimental enclosures, as changes in OM mineralization have been most pronounced in the surface and intermediate layers of peat (12, 24, 27). Following 3 years of *in situ* incubation, we measured changes in peat soil mass and chemical composition and characterized microbial communities in the decomposition bags through amplicon sequencing and network analyses. We hypothesized that decomposition of peat soil would increase with increasing temperature, driven by changes in their microbial communities.

## MATERIALS AND METHODS

### Site description

The SPRUCE experiment is located on the S1 bog (low pH, acid organic soil environment) at the USDA Forest Service Marcell Experimental Forest, MN, USA. The SPRUCE site description, experimental design, warming, and CO_2_ treatments have been previously described in detail (28). Briefly, above- and belowground, whole-ecosystem warming has been applied in a regression-based design to 10 open-air enclosures on the S1 bog since August 2015. Enclosures are duplicated for each level of warming (+0°C, +2.25°C, +4.5°C, +6.75°C, and +9°C above ambient), and half of the enclosures receive an elevated CO_2_ atmosphere (+500 ppm).

### Peat ladder construction, deployment, and retrieval

Organic soil used in the decomposition ladders was collected from within the S1 bog, but outside of the footprint of the actual experimental SPRUCE enclosures. Soil was carefully excavated using trowels and hand tools from four depths (0–10, 10–20, 20–30, and 30–40 cm), brought back to the laboratory, and air dried. After air drying, soil from each depth was separately homogenized by breaking up the dried soil using sieves, and large fragments of vegetation (i.e., roots) were removed. Soil was then weighed to include 2.4–4.2 g air-dry weight peat corresponding to an approximate wet weight of 20 g depending on the depth of collection, and placed into fine-mesh 6.5 × 9.0 cm bags (7 µm mesh size), which were then heat sealed (29, 30).

The fine-mesh bags were placed into decomposition ladders that were made of acrylonitrile butadiene styrene plastic (Fig. S1 [design concept from J. P. Megonigal, personal communication). The pre-weighed bags (described above) were inserted to the window position in the ladder corresponding with the same depth of peat from their original collection, and the ladders closed with plastic fasteners. This design thus allows the ladders to be placed vertically in the peat profile to allow for depth-specific measurements of soil decomposition. Each ladder had four openings (6.5 cm wide by 9 cm tall) corresponding to the four soil depths (Fig. S1). Each ladder was constructed by placing four soil-filled fine-mesh bags, one from each depth, between two plastic ladder holders and the ladders also lined with mesh (0.5 × 1.0 mm mesh size) to reduce abrasion of the fine-mesh bags during deployment and retrieval.

The pre-constructed ladders were deployed on 2 October 2017 randomly into the 10 SPRUCE enclosures and 2 ambient (unchambered) control plots. Ladders were placed vertically into the peat so that the peat collected from 0 to 10 cm was incubated at that same depth. Three replicate ladders were deployed per enclosure per planned retrieval time (12 in total per enclosure) and were placed in three different hollow locations that are associated with companion litter and wood decomposition studies. Three of these 12 replicate ladders per enclosure were deployed and then immediately retrieved from the peatland for measurement of initial mass, as well as carbon, nitrogen and phosphorus content (t = 0 y; T_0_). On 15 October 2020 (t = 3 y; T_f_), three replicate ladders per enclosure were retrieved to measure soil mass loss, changes in C/N/P, FTIR-based chemical composition, and microbial community changes over the 3 years of deployment (the data reported here). Additional ladder retrievals and measurements at years 6 and 10 are planned in the future and will be reported elsewhere.

### Peat soil mass loss and carbon, nitrogen, and phosphorus analyses

After retrieval, ladders were placed into individual plastic bags and were stored at 4°C until processing (within 24 hours). Next, each fine-mesh bag was carefully removed from the ladder, and the soil was weighed to determine wet mass. For the T_f_ bags, soil from each mesh bag was sub-sampled so that ~1/2 was retained for dry mass and chemistry measurements, ~1/4 was frozen at −20°C for microbial community analyses, and the remainder was frozen (−20°C) for archival purposes. Soil collected at T_0_ was not sub-subsampled as only dry mass and chemistry measurements were conducted.

Soil samples for mass loss and chemistry were air dried in a drying room (humidity <30%) for 1–2 months (until the change in mass was <5%) and then weighed. A sub-sample from each T_f_ sample was oven-dried to calculate an air-dry to oven-dry conversion factor and to calculate percent peat mass loss on an oven-dry basis. Peat used in construction of decomposition bags were not oven-dried prior to installation (T_0_) into the enclosures to prevent changes in organic matter quality; however, initial masses were corrected based on the air-dry to oven-dry conversion calculated from each sample at T_f_. Each air-dried sample from T_0_ and T_f_ was ground (IKA Tube Mill grinder, Wilmington, NC, USA), and a sub-sample was analyzed for carbon and nitrogen content using a combustion elemental analyzer (LECO-CHN628 analyzer, St. Joseph, MI, USA).

### FTIR analysis

Fourier transform infrared spectroscopy (FTIR) analysis was performed on the peat ladders to analyze the response of peat soil organic carbon fractions to enclosure treatments. The peat soil was ground into a homogenous powder using a Spex Sampleprep 5100 Mixer-Mill. FTIR spectra were collected using a JASCO 6800 FT-IR Spectrometer. Approximately 0.003 g of sample powder was secured onto the quartz crystal (Si/CaF2), and infrared light from wavenumbers 4,000 cm^−1^–650 cm^−1^ was transmitted onto the sample at a resolution of 4 cm^−1^. Each spectrum was attenuated, total reflection corrected, and baseline corrected to account for variability in the beam penetration depth. To produce the functional data for molecular composition analysis, eight spectra per sample were averaged.

The spectra data were analyzed using Hodgkins’ normalization method (31). Instrument and matrix variation impact on sample spectra absorbance were accounted for by dividing the baseline-corrected peak heights by the total integrated area of the spectrum. Using the maximum baseline-corrected absorbance between peak endpoints, the aromatics and carbohydrates functional group locations were identified. The normalized aromatics spectral peak heights were located at 1,510 cm^−1^ and 1,615 cm^−1^. The normalized carbohydrate spectral peak height was at 1,040 cm^−1^. Each of these peak heights was used to calculate the percent of aromatics and carbohydrates in each sample.

### DNA extraction and sequencing

DNA was extracted from ~0.2 g of field-wet peat soil from all samples using the Omega Biotech (Norcross, GA, USA) 96-well DNA Extraction Kits following the manufacturer’s protocol, which resulted in improved yields and quality DNA from the peat substrate compared to previous methods employed in our lab (12, 18). Amplicon metagenomic sequencing libraries were prepared as described in the Illumina 16S metagenomic sequencing library preparation guide (Part 15044223 Rev B) with a custom mixture of 515F and 806R primers for archaea/bacteria targeting the 16S rRNA gene and primers designed to the ITS2 spacer region within the rRNA region for fungi as we have reported previously (32, 33). Pooled libraries for each sample type were validated on an Agilent Bioanalyzer (Agilent, Santa Clara, CA) using a DNA7500 chip, and the final library pool concentration was determined on an Invitrogen Qubit (Waltham, MA) with the broad range double stranded DNA assay. Paired-end sequencing (2 × 251 × 8 × 8) was completed on an Illumina MiSeq instrument (Illumina, San Diego, CA) using v2 chemistry. Due to low base diversity of the amplicons, PhiX control DNA was included in the sequencing run.

### Sequence analysis

Samples were demultiplexed, and paired-end 16S rRNA (V4 region) and ITS2 sequences were assembled using standard Illumina software and protocols and exported for analyses in QIIME2 (version 2021.4) (34). ITS2 primer sequences were removed using the cutadapt plugin (35), and 16S rRNA gene primers were removed using the dada2 plugin (36). All sequencing runs were individually denoised and ASVs identified using the dada2 plugin (36). Resulting feature tables and representative sequences were merged for downstream analyses. Taxonomy was assigned using the silva database (16S; version 138) (37) and UNITE database (ITS2; version 8.0) (38). Sequences and feature tables were filtered based on taxonomic assignments to include only bacteria and archaea, removing chloroplast and mitochondria sequences (16S) and fungi with taxonomy assigned to the phylum level at a minimum (ITS2). A rooted phylogenetic tree was built for each data set using the align-mafft-to-fasttree pipeline (39) in the phylogeny plugin.

### Statistical analyses

All statistical analyses and figures were produced in R version 4.1.0 with the vegan (40), car, phyloseq (41), ggplot2 (42), microbiome, SpeicEasi (43), igraph (44), and hilldiv (45) packages. ITS and 16S feature tables, taxonomy, rooted phylogeny, and associated metadata were imported and analyzed as phyloseq objects. Based on the regression design of SPRUCE, temperature treatment (+0°C, +2.25°C, +4.5°C, +6.75°C, and +9°C) was used as a continuous variable for all statistical analyses. Soil depth and CO_2_ treatment were treated as categorical variables, and depth-specific subsets of the data were generated to investigate the effects of temperature and CO_2_ at a given depth. Samples were rarefied for α-diversity analyses (16S rarefaction depth = 9,500; ITS2 rarefaction depth = 1,000). The hilldiv package was used to calculate α-diversity metrics as Hill numbers (effective number of species) (46), and two-way ANOVA analysis was used to investigate the effects of temperature, soil depth, CO_2_ treatment, and the interaction between temperature and depth on α-diversity. Bray-Curtis dissimilarities (47) of total sum scaled data were calculated and used to assess the effects of temperature, depth, CO_2_ treatment, and the interaction between temperature, CO_2_, and soil depth on community composition by PERMANOVA. Principal coordinates analysis plots of Bray-Curtis dissimilarities were generated to visualize community composition. The betadisper function was used to assess differences in β-dispersion across depths, temperature treatments, and CO_2_ treatments.

Trans-domain networks were generated using the SpiecEasi and iGraph packages in R. Networks were constructed for each temperature treatment and included all depths within a given temperature treatment (*n* = 24 network^−1^). Phyloseq objects were filtered by temperature treatment, and only amplicon sequence variants (ASVs) with a total sum of >5 (16S) or >3 (ITS2) and an occurrence in >20% (5/24) of samples were included in the network. If a sample was lacking either a 16S or ITS2 library after filtering, both libraries from that sample were excluded from analysis (12 samples were excluded across 120 total samples). Network parameters were set as method = mb, nlambda = 50, lambda.min.ratio = 1e-3, and thresh = 0.01. Empty nodes were removed, and networks were visualized using phyloseq. The number of nodes, edges, node degree, and betweenness centrality were calculated for each network with the igraph package. Network hubs were identified by selecting nodes that had degree and betweenness centrality measures in the 90th percentile, indicating high connectedness and centrality in the network. Pearson correlation was used to investigate the relationship between network topology and SPRUCE temperature treatments. The identity of ASVs of prominent network hub taxa was verified by the analyses of closely matching sequences in BLAST searches (48), and in the case of fungal taxa, their potential functional roles were investigated using FUNGuild (49).

Kruskal–Wallis test was used to compare peat soil mass and chemical composition at T_0_ to T_f_. Effect sizes for T_0_-T_f_ comparisons were calculated as (χ^2^ - 1)/(*n* - 2), where χ^2^ is the Kruskal–Wallis test result and *n* is the number of samples. Linear models were used to assess the effect of depth, temperature treatment, and CO_2_ treatment on soil mass loss and chemical compositional changes.

## RESULTS

### Community summary

A total of 9,962 bacterial/archaeal ASVs and 3,302 fungal ASVs were observed across all samples. Bacterial/archaeal communities were dominated by the phylum *Acidobacteriota* across soil depths and temperature treatments (28%–46% mean relative abundance) (Fig. S2). Near the surface (0–10 cm and 10–20 cm), *Actinobacteriota*, *Proteobacteria*, and *Planctomycetota* comprised approximately 35%–45% of the bacterial/archaeal communities, while accounting for <20% of the total community at 20–30 cm and 30–40 cm (Fig. S2). Dominant phyla of the fungal communities also varied across depth, with *Basidiomycota* in highest relative abundance at 0–10 cm (~62% average relative abundance), and *Ascomycota* dominating in the deeper three depths (~54-68% average relative abundance) (Fig. S3).

### Microbial community composition and α-diversity are significantly influenced by temperature and CO_2_ treatments

**Fig 1:**
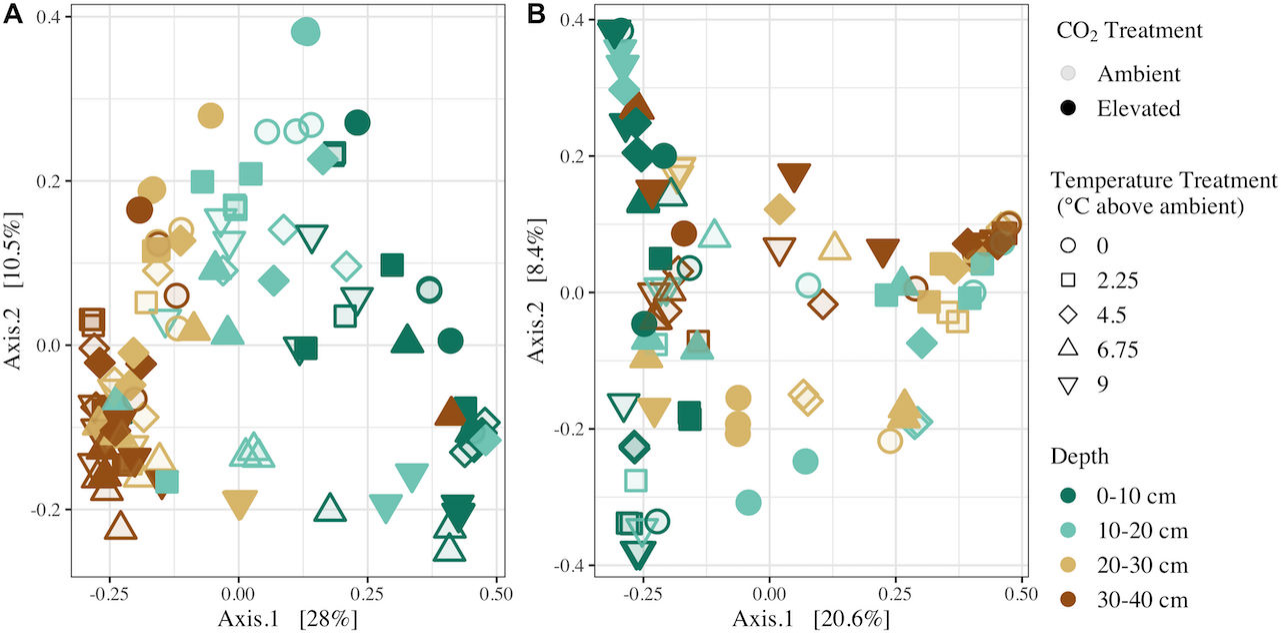
Principal coordinates analysis of Bray-Curtis dissimilarities of bacterial/archaeal (**A**) and fungal (**B**) communities in decomposition ladders. Points are colored based on sample depth, filled based on enclosure CO_2_ treatments, and shaped based on enclosure temperature treatments.

Bacterial/archaeal and fungal community compositions were significantly impacted by depth, temperature treatment, CO_2_ treatment, and the interactive effects of these variables (Fig. 1; Table 1). Of the variables investigated, depth was the most influential factor driving compositional differences in the bacterial/archaeal and fungal communities, explaining 25% and 11% of the variation, respectively (Table 1). Temperature treatment explained 6.1% of the variation in the bacterial/archaeal community and 4.9% in the fungal community, and CO_2_ treatment accounted for 1.9% and 2.7% of the variation for both bacterial/archaeal and fungal communities, respectively (Table 1). Significant interactive effects between soil depth and temperature treatment, and between temperature treatment and CO_2_ treatment were observed for the bacterial/archaeal communities (Table 1). Significant interactions between all tested variables were observed for fungal communities, such that temperature and CO_2_ treatment only influenced fungal community composition at specific depths (Table 1). No significant differences in β-dispersion were observed across soil depth, temperature treatment, or CO_2_ treatment.

**TABLE 1:** Results of permutational multivariate analysis of variance (PERMANOVA) of bacterial/archaeal and fungal community compositions based on Bray-Curtis distances^*a*^

Variable	Bacterial/archaeal			Fungal		
	F-statistic	R^2^	*P*-value	F-statistic	R^2^	*P*-value
Depth	15.402	0.25323	**0.001**	4.0315	0.10959	**0.001**
Temperature treatment	11.1683	0.06121	**0.001**	5.46	0.04948	**0.001**
CO_2_ treatment	3.3775	0.01851	**0.004**	3.0053	0.02723	**0.001**
Depth:temperature treatment	2.907	0.03766	**0.001**	1.9049	0.05178	**0.001**
Depth:CO_2_ treatment	1.2092	0.01988	0.176	1.6339	0.04442	**0.006**
Temperature treatment:CO_2_ treatment	4.983	0.02731	**0.001**	2.6901	0.02438	**0.004**
Depth:temperature treatment:CO_2_ treatment	1.4101	0.02318	0.069	1.8305	0.04976	**0.001**

^*a*^ Significant factors and interactive effects (*P* < 0.05) are bolded.

**Fig 2:**
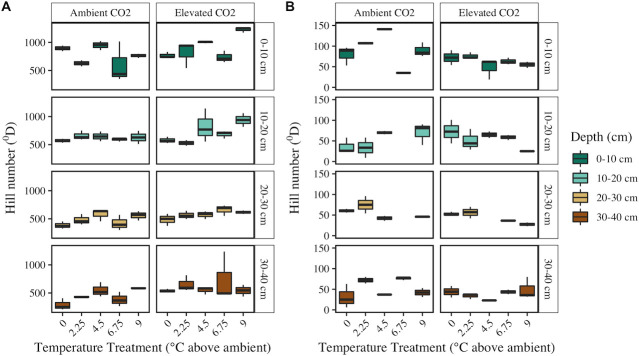
Bacterial/archaeal (**A**) and fungal (**B**) α-diversity at q = 0 (species richness) across depths (vertical facets/colors), CO_2_ treatments (horizontal facets), and temperature treatments (x-axis). Note that the PCR and sequencing runs for three fungal samples failed to produce a minimum number of quality controlled sequences and were thus omitted from analyses and this figure (panel B, second and third rows).

α-diversity trends were measured using Hill number approaches, where species richness is equivalent to q = 0, Shannon entropy equivalent to q = 1, and inverse Simpson equivalent to q = 2. Bacterial/archaeal α-diversity was highest near the surface and declined with depth across all three levels of q (Fig. 2A; Fig. S4; Table 2). Temperature treatment also significantly impacted bacterial/archaeal α-diversity, with highest diversity measured in decomposition ladders from the +9°C enclosures across all α-diversity metrics (Fig. 2A; Fig. S4; Table 2). Bacterial/archaeal α-diversity was also significantly higher in elevated CO_2_ enclosures when compared to ambient CO_2_ at q = 0 and q = 1 (Fig. 2A; Fig. S4A; Table 2). At q = 2, no effect of CO_2_ on α-diversity was observed, suggesting that CO_2_ treatment has a limited effect on abundant bacterial/archaeal taxa (Fig. S4B; Table 2). In contrast, fungal α-diversity was significantly lower in enclosures with elevated CO_2_ when compared to ambient CO_2_ enclosures across all α-diversity metrics measured (Fig. 2B; Fig. S5; Table 2). Soil depth only significantly impacted fungal species richness (q = 0), with highest richness observed at 0–10 cm, and we observed a significant effect of temperature treatment at q = 2 (Fig. 2B; Fig. S5; Table 2) and significant interactive effects of temperature and depth at q = 1and q = 2, such that temperature effects on fungal α-diversity were depth specific.

**TABLE 2:** Resulting *P*-values from ANOVA describing the effects of depth and SPRUCE treatments on bacterial/archaeal and fungal α-diversity across Hill numbers (q = 0, q = 1, and q = 2)^*a*^

Variable	16S			ITS		
	q = 0	q = 1	q = 2	q = 0	q = 1	q = 2
Depth	**<0.001**	**<0.001**	**<0.001**	**<0.001**	0.802	0.296
Temperature treatment	**0.002**	**<0.001**	**<0.001**	0.728	0.113	**0.034**
CO_2_ treatment	**<0.001**	**0.015**	0.133	**0.009**	**<0.001**	**0.002**
Depth:temperature treatment	0.978	0.907	0.856	0.094	**0.013**	**0.003**

^*a*^ Significant values (*P* < 0.05) are bolded.

### Trans-domain networks

**Fig 3:**
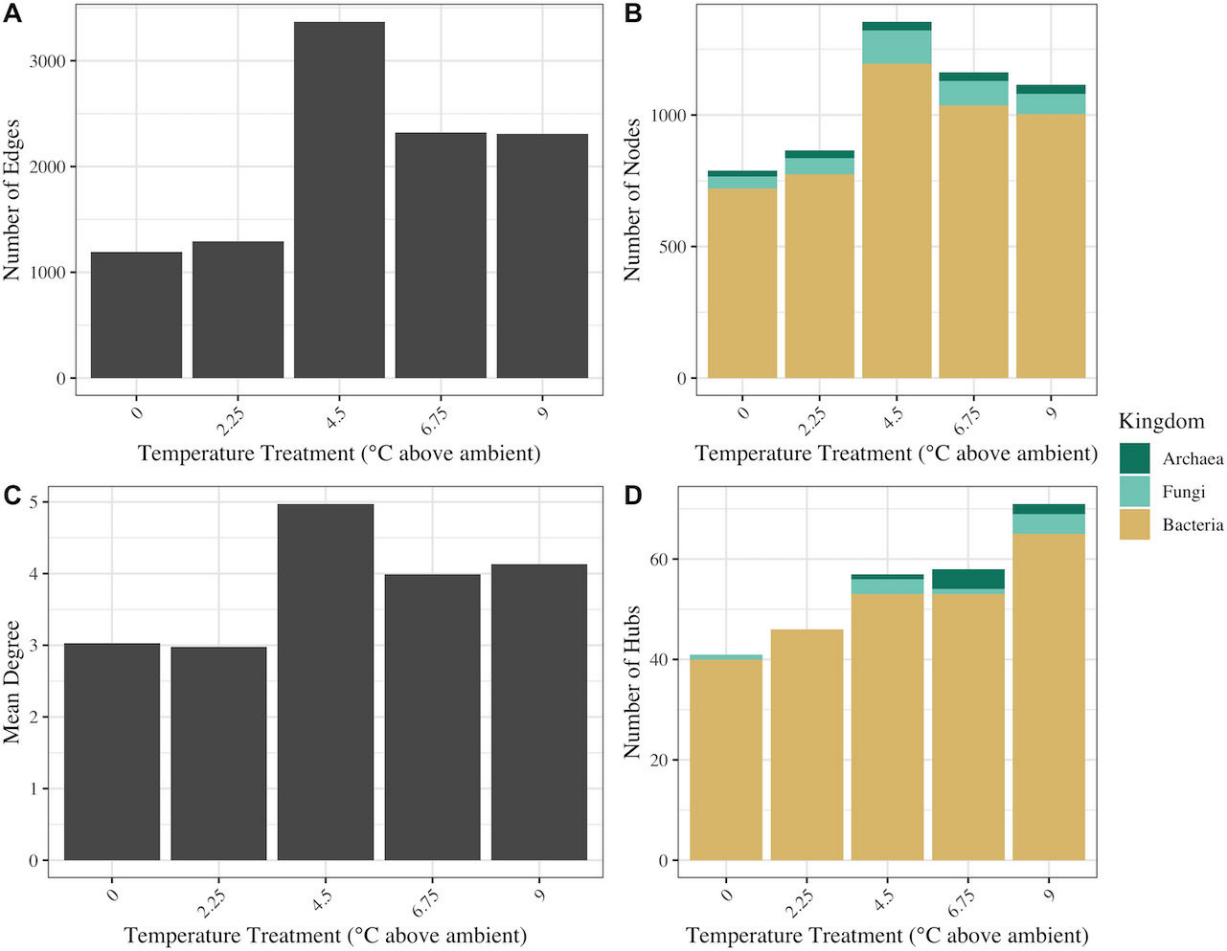
Summary of network topologies for temperature treatment microbial networks. The number of edges (**A**) represents the sum total connections between taxa (nodes; **B**) within the network. Mean degree (**C**) represents the average number of connections per taxon. Hubs (**D**) are defined as nodes within the network that are in the 90th percentile for degree and betweenness centrality and represent highly connected and centralized taxa.

We constructed temperature treatment-specific, trans-domain networks to investigate the impact of temperature treatment on the connectedness of microbial communities through comparisons of network topology (Fig. S6). The microbial network from +0°C enclosures across all depths had the lowest number of nodes (taxa), edges (connections between taxa), and average degree (mean number of edges per node), collectively indicating lower complexity of the microbial communities in +0°C enclosures when compared to warmed enclosures (Fig. 3). The number of nodes and edges and the average degree peaked in the network built from +4.5°C enclosures (Fig. 3A, B and C). Networks built from all the temperature treatments included bacterial, archaeal, and fungal nodes, with the highest number of archaeal nodes present in the +9°C network (35 nodes), and the most fungal nodes occurring in the +4.5°C network (126 nodes) (Fig. 3B).

**Fig 4:**
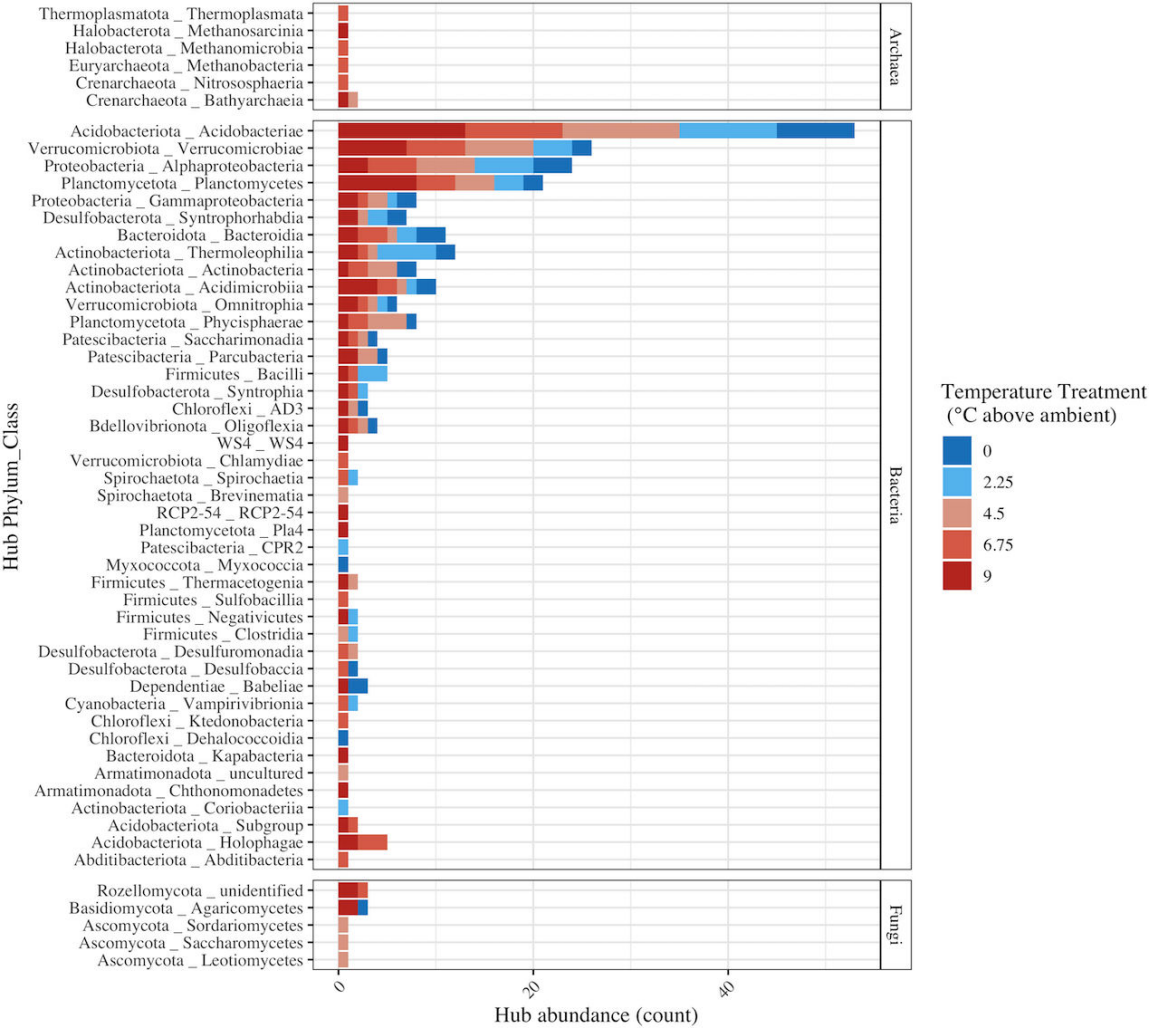
Class-level distribution of network hubs across temperature treatments (color). Hubs were aggregated at class-level taxonomic annotations (y-axis) and faceted by kingdom-level taxonomy.

Within each temperature treatment network, we identified hub taxa as those with degree and betweenness centrality measures in the 90th percentile. The number of hub taxa was significantly correlated with temperature (*R^2^* = 0.975, *P* = 0.005), with a high of 71 hubs in the +9°C network (Fig. 3D). Archaeal hubs were present in the +4.5°C, +6.75°C, and +9°C networks, and fungal hubs were observed in all networks except the +2.25°C network (Fig. 3D). Class-level taxonomic distribution of the hub taxa revealed that *Acidobacteriae* were the most prominent across all treatments and depths, followed by the *Verrucomicrobiae* (Fig. 4). In the +9°C network, six unique classes were represented in the hubs, including the methanogenic class *Methanosarcinia* (Fig. 4). Two unique classes were present in the +0°C network hubs, *Myxococcia* and *Dehalococcoidia* (Fig. 4). Only three of the fungal hubs could be assigned to a guild by FUNguild analysis, including possible ectomycorrhizal and saprotrophic *Agaricomycetes* hubs and a *Sordariomycetes* hub that is a probable plant pathogen.

### Peat soil mass loss and chemical compositional changes are highly variable

**Fig 5:**
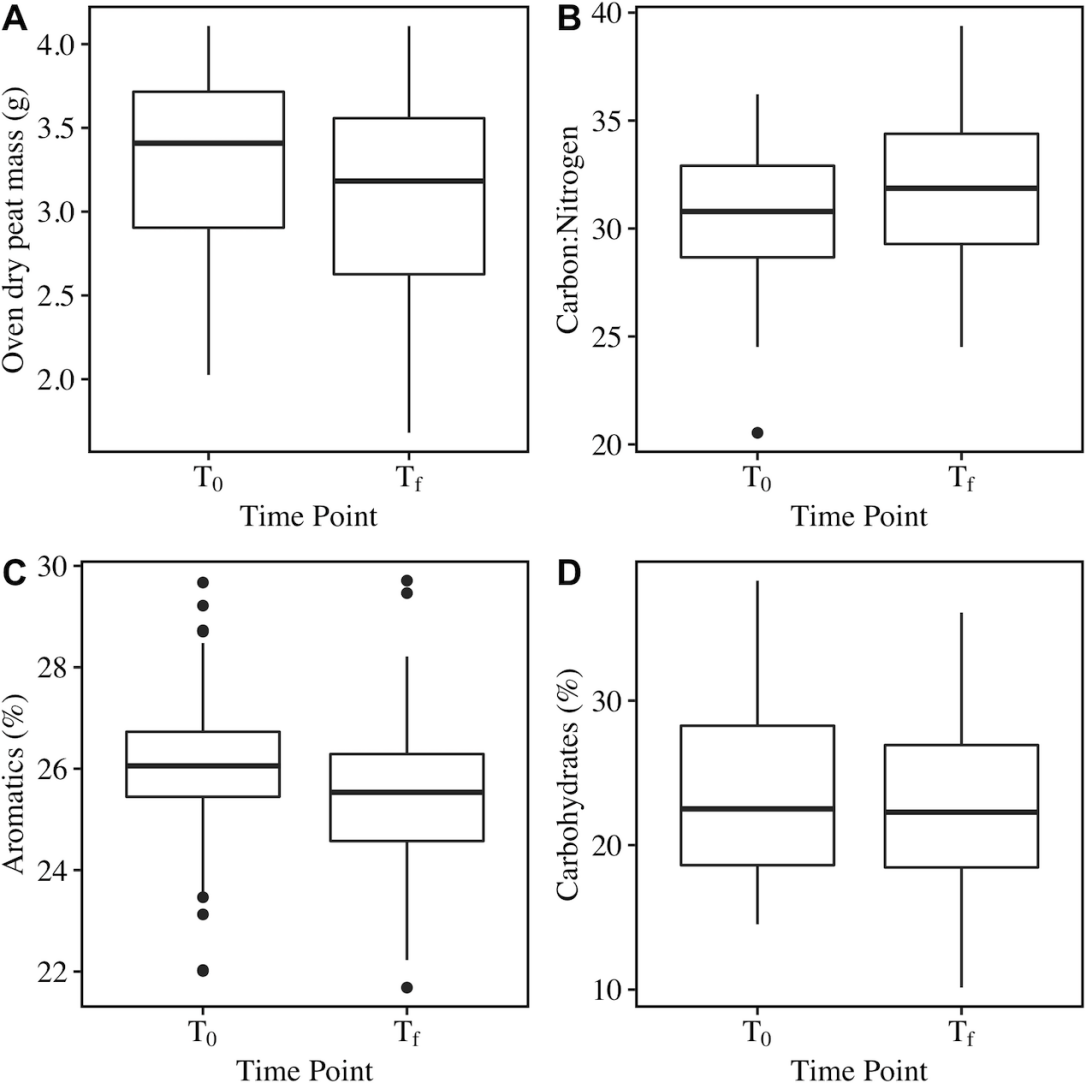
Comparisons of peat soil oven dry mass (**A**), carbon:nitrogen (**B**), percent aromatics (**C**), and percent carbohydrates (**D**) in peat decomposition ladders at the start of the experiment (**T_0_**) and after 3 years of incubation in the SPRUCE enclosures (T_f_). Box and whisker plots display the median (middle of box), quartiles (top and bottom of box), minimum and maximum values excluding outliers (end of whisker), and outliers (points).

**Fig 6:**
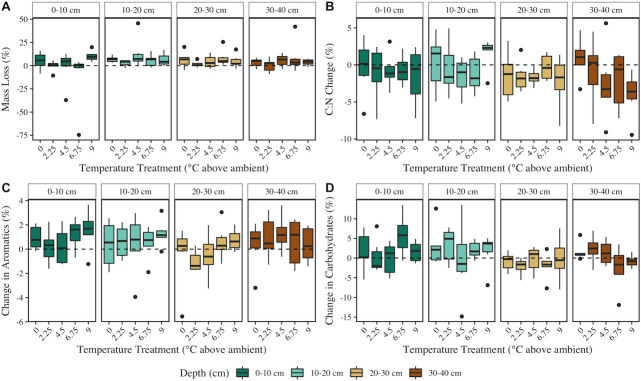
Peat mass loss (% of starting mass lost) (**A**), percent C:N change (T_f_ – T_0_) (**B**), percent aromatics change (T_f_ – T_0_) (**C**), and percent carbohydrates change (T_f_ – T_0_) (**D**) across depths (facets) and temperature treatments (x-axis).

On average across all depths and treatments, the mass of peat soil in decomposition ladders was significantly lower after 3 years of treatment (x̅ ± SD mass loss = 4.50% ± 11.07%; Kruskal–Wallis χ^2^ = 6.7158, *P* = 0.0095) (Fig. 5A); however, no significant effects of depth, temperature, CO_2_ treatment, or their interactions on peat soil mass loss were observed (Fig. 6A; Table S1). Peat C and N content decreased significantly over the course of the experiment (Fig. S7), and C:N of the peat was significantly higher at T_f_ compared to T_0_ (Kruskal–Wallis χ^2^ = 7.0841, *P* = 0.0078) (Fig. 5B), indicating a more rapid loss of N compared to C. Similar to soil mass loss, neither depth nor the treatment variables had a significant effect on the change in C:N (Fig. 6B; Table S1).

Results of Fourier-transform infrared spectroscopy analyses further showed limited effects of the treatments on peat soil composition after 3 years of incubation. Aromatics (%) decreased significantly over the course of the experiment (Fig. 5C), but there was no significant effect of soil depth or treatment on the change in aromatics (Fig. 6C; Table S1). Depth significantly impacted the change in % carbohydrates, with the largest change in the % carbohydrates at 0–10 cm. However, there was no significant difference in % carbohydrates between T_0_ and T_f_ (Fig. 5D), and temperature and CO_2_ treatment did not significantly impact carbohydrate content (Fig. 6D; Table S1).

## DISCUSSION

The effects that climate change will have on peatland microbial communities and the resulting impacts on soil decomposition have yet to be fully resolved but could have outsized effects due to the massive stores of carbon in peatlands. Previous results from SPRUCE have shown rapid loss of carbon presumably driven by increased decomposition in response to elevated temperature (10). In this study, we utilized decomposition ladders to investigate the effects of warming and elevated CO_2_ on peat microbial communities and decomposition while limiting inputs from primary productivity. Our results show that bacterial/archaeal and fungal communities are significantly impacted by the SPRUCE treatments; however, in contrast to previous research, we did not observe a significant effect of temperature, elevated CO_2_, or soil depth on peat mass loss or chemical composition changes. An average of less than 4.5% of the initial mass was lost over the 3-year experiment regardless of treatment, demonstrating the high recalcitrance of organic soils in peatlands, and suggesting that these prior reported results may driven by turnover of more recently fixed C rather than the historic C stocks of peat studied here. These small differences also illustrate just how inherently difficult measure such decomposition is to measure given unavoidable experimental variability between replicates and other sources of error, combined with small mass losses of these recalcitrant substrates.

Bacterial/archaeal and fungal community compositions were significantly influenced by the SPRUCE treatments (Fig. 1; Table 1), indicating that increased temperature and atmospheric CO_2_ as a result of climate change may alter microbial ecology in peatlands. Consistent with previous research, bacterial/archaeal community responses to temperature treatment were more pronounced than fungal responses (22, 50, 51). Bacterial/archaeal α-diversity was significantly highest in the warmest SPRUCE enclosures across all soil depths, whereas the influence of temperature treatment on fungal α-diversity was depth specific and only observed at q = 2 (Fig. 2; Table 2). Ecosystem disturbance and environmental stress have been negatively correlated with microbial diversity (52); thus, higher bacterial/archaeal diversity in warmed enclosures suggests that warming may alleviate environmental stress on prokaryotic communities in peatlands. Higher bacterial/archaeal diversity may be a direct cause of warming; however, contrasting results have also been observed in anaerobic peat soil microcosms that investigated direct warming effects (17, 21). Indirect effects such as warming-induced increases in substrate and nutrient availability (53) are therefore more likely driving changes in bacterial/archaeal diversity by partially relieving nutrient stress. Our experimental design did not allow us to delineate between direct and indirect effects of warming, as porewater total organic carbon and nutrient concentrations were largely correlated with temperature treatment on average over the course of the experiment (Table S2). However, previous research has demonstrated that labile substrate and nutrient availability and microbial diversity are positively correlated which supports our results.

Network analyses further revealed the impact of increased temperature on peat microbial community structure. The number of nodes and edges were highest in the networks from warmed enclosures (Fig. 3), and a positive correlation between the number of input ASVs and edge and node counts indicates that this is likely a reflection of species richness. Species richness and changes in network topology such as a decreased ratio of positive to negative edges and increased modularity in microbial networks have been shown to correspond to higher community stability (52, 54, 55), potentially through increased microbial functional redundancy (56). Paired with diversity measures, our network results indicate that warming may positively influence microbial community stability.

The abundance of microbial hub taxa (those taxa which are highly connected within the network) was positively correlated with temperature treatment (Fig. 3D). The number of hub taxa within microbial networks has been associated with functional potential of the microbial community (57, 58), and hub taxa may exert strong influence over microbiome structure and ecosystem function regardless of their relative abundance within the community (59, 60). Therefore, our results suggest that warming may promote increased microbial functional potential in these peatland ecosystems.

We observed *Methanomicrobia*, *Methanobacteria*, and *Methanosarcinia* hubs in the +6.75°C and +9°C networks (Fig. 4), supporting results that show methanogenesis is an increasingly important function in peatland carbon-cycle response to warming. The relative abundance of methanogenic taxa has been shown to increase in response to increasing temperature in incubations (17), and rates of methane production have largely been shown to increase with warming (61), including in incubation studies of soil from the S1 bog (17). Detection of an acetoclastic methanogen in the +9°C network corresponds to isotopic analysis of CH_4_ in the SPRUCE enclosures indicating that acetoclastic methanogenesis is increasing with warming (61). A significant linear relationship between *in situ* porewater concentrations of CH_4_ and temperature treatment has been observed in the top 25 cm of soil at SPRUCE (12), further supporting our results. Methane has a global warming potential of 28 times that of CO_2_ on a 100 years time span (6), and higher potential for methanogenesis in response to climate change may fuel a positive feedback loop.

Methanogenesis can be supported by syntrophic interactions, especially under nutrient-limiting conditions that are observed in bogs (62, 63). Two known syntrophic taxa, *Syntrophia* (64) and *Syntrophorhabdia* (65), were identified as hubs within the networks (Fig. 4), suggesting the potential importance of syntrophy within the sites. Other hubs identified in the networks from warmed enclosures including *Bathyarchaeia* and *Holophagae* may further support increased acetoclastic methanogenesis, as these taxa have been previously shown to have the potential for acetogenesis in anoxic environments (66, 67).

Studies of peatland responses to disturbance often investigate fungal and bacterial/archaeal communities independent of one another despite knowledge of the complex interplay across microbial domains (68, 69). Here, we observed the highest number of fungal nodes and hub taxa in networks from decomposition ladders in warmed enclosures, suggesting that peatland warming may promote trans-domain interactions, further arguing for such holistic approaches. Possible saprotrophic and ectomycorrhizal fungal hub taxa were primarily observed in the networks from warmed enclosures apart from an *Agaricomycetes* hub in the +0°C network (Fig. 4). Fungal communities play an important role in OM decomposition in peatlands (70), and warming may favor dominance of saprotrophic and mycorrhizal fungi from *Basidiomycota* and *Ascomycota* (71), as partially observed in our study. Additionally, warming treatments in these systems are inextricably linked to drying of the peat surface and increased depths to the water table. It is thus possible that peat drying and water table changes, more so than warming, may be responsible for the shifts in fungal communities observed; however, the average water table height was similar across all SPRUCE enclosures in the week prior to termination of the experiment (0.17 m ± 2.47 cm) as well as the 3 prior months and there were no significant effects on peat water content in the decomposition bags at the time of harvest. However, this does not rule out that moisture was not a significant factor throughout parts or even the majority of the 3-year period of the decomposition experiment, only that we did not observe such effects based on data at near the time of harvest.

In contrast to bacterial/archaeal diversity, fungal α-diversity was significantly lower under elevated CO_2_ compared to ambient. Fungal responses to CO_2_ treatment are likely mediated by plant responses, as CO_2_ treatment at SPRUCE is above-ground and is unlikely to directly alter soil biogeochemistry. Interactions between plant roots and fungi are common, and fungi are most prevalent (absolute abundance) near the surface of the peatland where active plant growth occurs (72). Our results are intriguing and suggest further investigation into plant-fungal interactions under elevated CO_2_ conditions.

Significant changes in the peat microbial communities in response to SPRUCE treatments were not mirrored by the peat soil decomposition rates. We anticipated that increased temperature would result in increased peat soil mass loss, as temperature treatment at SPRUCE has resulted in rapid carbon loss that was presumed to be driven by enhanced decomposition (10), increased CO_2_ and CH_4_ in porewaters (12), and increased microbial respiration of solid phase peat (13). However, our results showed that soil mass loss and C:N were not significantly impacted by temperature or CO_2_ treatment over the course of 3 years (Fig. 6). The lack of differences is likely driven by the short time scale and low initial mass of peat soil in the decomposition bag study compared to the SPRUCE enclosures. Hanson et al. (10) estimated the rate of carbon loss from SPRUCE to be 31.3 g C·m^−2^·year^−1^· °C^−1^ using approaches including elevation changes and ecosystem CO_2_ flux mass balances. Using this rate to estimate the expected loss of carbon from the peat decomposition ladders suggests that differences in mass loss across temperature treatments were on the order of milligrams over a 3-year period, thus likely requiring a level of precision that we were unable to obtain in our litter bag-based experiments.

On average across all depths and treatments, only 4.5% of the initial peat soil mass was lost over the course of the experiment (Fig. 5). The low mass loss demonstrated the recalcitrance of the organic soils in the decomposition ladders is likely driven by a combination of factors including the anoxic, acidic, and oligotrophic conditions of the sites, as well as the chemical composition of the peat. Peat soils at SPRUCE are largely derived from *Sphagnum*, which is known to engineer acidic, nutrient poor, waterlogged conditions (73, 74) and produces anti-microbial compounds and metabolites (75, 76), thereby inhibiting microbial degradation processes. Even the mass loss of fresh *Sphagnum* litter in decomposition bags has been previously shown to be similarly low with only ~10% of initial mass lost after 2 years (77, 78), so these lower rates for peat soil should not be unexpected.

Diffusion of exogenous dissolved organic matter (DOM) into the decomposition ladders may have also helped explain the differences in results between mass loss and community change. Dissolved organic matter is preferentially mineralized by peatland microorganisms when compared to solid-phase peat (79), and fresh plant inputs of DOM can even fuel microbial respiration in deep peat soils (80). Utilization of exogenous DOM may also partially explain discrepancies between our results and previous results from SPRUCE that have shown increased CO_2_ and CH_4_ production with warming. Porewater concentrations of total organic carbon were highest in the +9°C enclosures near the termination of our experiment, and higher inputs of DOM may have led to increased OM mineralization and shifts in microbial community structure without impacting peat soil mass in the decomposition ladders.

Similar to soil mass-loss observations, FTIR analysis indicated that temperature and CO_2_ treatment had no effect on changes in peat soil composition. The relatively short duration of the experiment may have masked temperature effects on the percent aromatics and carbohydrates of the peat, although previous results have indicated that carbon at SPRUCE is compositionally stable (81). We are hopeful that our future planned ladder extractions at our site with their longer field incubation periods may allow for better assessment of treatment effects on peat soil decomposition.

### Conclusions

While we did not observe changes in peat soil mass or composition across the SPRUCE treatments in this study, previous research from the SPRUCE experiment has shown loss of OM near the surface and increased greenhouse gas production in response to elevated temperatures. Our results suggest that these losses in OM may be driven by changes in microbial community structure and dynamics, as we observed significant changes in microbial diversity and network structure in response to warming. The apparent decoupling of changes in peat soil mass and composition and microbial communities may be limited by the very slow peat decomposition rates and precision of mass loss estimates in our study. Collectively, our results and previous results from the SPRUCE experiment therefore suggest that climate change may alter peatland microbial ecology however the ultimate effects of these changes on rates of degradation of OM and greenhouse gas production in boreal regions remains unclear at this time.

## Data Availability

Amplicon sequences generated in this study have been deposited in the NCBI SRA under BioProject ID PRJNA941900. The full peat decomposition and chemistry data set is deposited as part of our SPRUCE data archive: https://doi.org/10.25581/spruce.111/1991516. All code used in statistical analysis of the data and generation of figures is available on GitHub at https://github.com/swroth/Peat_Decomp_SPRUCE.

## SUPPLEMENTAL MATERIAL

Supplemental material msystems.00337-23-s0001.docx Supplemental figures and tables.

ASM does not own the copyrights to Supplemental Material that may be linked to, or accessed through, an article. The authors have granted ASM a non-exclusive, world-wide license to publish the Supplemental Material files. Please contact the corresponding author directly for reuse.
